# Equal access, unequal outcomes? racial disparities in mortality between black and white women with breast cancer: a 10-year systematic review and meta-analysis

**DOI:** 10.1007/s11764-025-01791-8

**Published:** 2025-03-27

**Authors:** Aaron J. Hill, Naren Durbha, Yvonne L. Eaglehouse, Y. Alicia Hong, Hong Xue

**Affiliations:** 1 Murtha Cancer Center Research Program, Department of Surgery, Uniformed Services University of the Health Sciences, Bethesda, MD 20817 USA; 2 Department of Preventive Medicine & Biostatistics, F. Edward Hébert School of Medicine, Uniformed Services University of the Health Sciences, Bethesda, MD 20814 USA; 3 Department of Health Administration and Policy, College of Public Health, George Mason University, Fairfax, VA 22201 USA; 4 The Henry M. Jackson Foundation for the Advancement of Military Medicine, Inc, Bethesda, MD 20817 USA

**Keywords:** Breast cancer, Disparities, Health equity, Military health system

## Abstract

**Purpose:**

This study uses systematic review and meta-analysis to address whether the significant disparities in breast cancer (BC) related health outcomes between Black and White women in the U.S. exist for women treated in the equal access Military Health System (MHS).

**Methods:**

PubMed, CINAHL, and Web of Science were searched using PRISMA guidelines in February–March 2024 for articles published between 2014–2024 containing quantitative mortality outcomes for Black and White women with BC treated in the MHS. Summarized mortality risk was computed using random effects meta-analysis from 2 × 2 tables of total deaths and population. Search, study selection, and quality assessment were completed by two independent co-authors.

**Results:**

Six studies with samples ranging from 395 to 28,818 across 31 years of data were included, and their mortality findings synthesized. Compared to matched patients in the general population, both Black and White women treated in the MHS had reduced mortality risk in two studies. While unadjusted mortality risk varied across three studies with direct comparisons between Black and White women treated in the MHS, adjusting for demographic and cancer characteristics eliminated significant differences. Meta-analysis of all six studies revealed a higher pooled unadjusted mortality risk for Black compared to White women (OR: 1.20, 95% CI: 1.09–1.33).

**Conclusions:**

Equal access to health care alone may not eliminate racial disparities in mortality for patients with BC.

**Implications for Cancer Survivors:**

Improving access, in combination with other targeted interventions, may reduce disparities in health outcomes for women with BC.

## Introduction

Breast cancer (BC) is the second most common cancer in women of all ages in the U.S. and the second leading cause of cancer-related death [1]. Black women have a 41% higher risk of BC mortality than non-Hispanic White women in the U.S., despite having a 4% lower incidence rate [1]. In addition to social stress, environmental exposures, and genetic factors [2, 3], lack of access to care has been cited as one of the main contributors to the disparity in BC mortality [4, 5]. Insurance coverage in the U.S. is linked to employment status and other socioeconomic factors [6] while access to care may depend on insurance coverage, proximity to facilities, services offered in the community, ability to seek care, and trust in the health care system [7]. Differences in the U.S. population between Black and White persons in regards to these factors and historical experiences of discrimination may lead to disproportional access to medical care. Lack of insurance coverage has been shown to account for up to a third of the disparity in mortality between Black and White women with BC [8]. Lack of access has also led to disparities through significantly lower rates of mammography between Black and White women [9], which results in later stage presentation and increased mortality [10]. In addition to later stage presentation, disparities in mortality between Black and White women have been attributed to differences in treatment [11]. Comparing mortality among women with BC with equal access to screening and comprehensive treatment within the U.S. Military Health System (MHS) may offer additional insights into how access impacts mortality disparities among patients with BC.

The Department of Defense (DoD) operated MHS is one of the largest health systems in the U.S., caring for 9.6 M beneficiaries through the Direct Care (DC) and Private Sector Care (PSC) networks [12]. The DC network is comprised of nearly 129,000 personnel within over 700 hospitals and health clinics operated by the DoD [12]. The PSC network includes over 1 M affiliated providers caring for beneficiaries in the civilian community [12]. Beneficiaries include active-duty service members, family members, retirees from active military service, or others with approved access-to-care. Insurance coverage and access to both DC and PSC are provided regardless of the beneficiaries’ socioeconomic or demographic characteristics. Additionally, care delivered in the MHS is at low or no cost to the patient [13], which may reduce financial barriers to care experienced in the general population.

BC is the most common cancer in active duty service women (ADSW) [14, 15], that is women who are employed full-time within the U.S. military. Additionally, ADSW have been shown to have between 20 and 40% higher incidence of BC diagnoses, compared to women in the U.S. general population [16]. Analysis to support a 2024 report to Congress found no significant risk factors associated with military service that differed from those of the general population [14], which is consistent with previous research attributing the increased incidence to universal access and increased screening [16]. In the equal access MHS, consistency in care among patients treated for BC and higher survival than the U.S. population were noted [17]. In addition to increased survival, no racial disparities were noted in BC screening for patients within the MHS [18].

Whether racial disparities in mortality risk exist among women treated in the MHS remains inconclusive. The authors are aware of studies which include racial comparisons of mortality among women with BC in the MHS, which provide mixed results on the association between race and mortality [19, 20]. Examining racial differences in breast cancer mortality in the MHS will provide information on whether disparities persist under conditions of equal access to care. If substantial disparities remain, it points to factors other than access to care, such as biological or social factors, and their role in racial disparities in BC mortality. The aim of this study is to expand on this knowledge and conduct a systematic review of the literature and provide a meta-analysis of pooled risk of mortality comparing Black and White women with BC treated in the MHS.

## Methods

### Search strategy

A systematic literature review following the Preferred Reporting Items for Systematic Reviews and Meta-Analyses (PRISMA) guidelines was conducted [21]. As shown in Table 1, PubMed, CINAHL, and Web of Science were searched in February and March 2024, using key words for articles published between 2014 and 2024 containing quantitative mortality outcomes for Black and White women with BC treated in the MHS. Since racial-ethnic terminology varied among studies, relevant papers were identified using African American, non-Hispanic Black, Black and European American, non-Hispanic White, Caucasian, and White. For consistency, the racial-ethnic groups represented by these terms are hereafter referred to as “Black” and “White”, respectively. Key words consisted of Boolean searches containing combinations of “Department of Defense”, “Military Health System”, “military hospitals”, “breast cancer”, “breast neoplasms”, “survival”, “mortality”, and “death” (Table 1).

**Table 1:** Search strategy, 2014 to 2024 studies of mortality comparing Black and White Women with breast cancer treated in the MHS

**Database**	PubMed	CINAHL	Web of Science
Search Terms	((("United States Department of Defense"[Mesh]) OR (department of defense[Title/Abstract]) OR ("Hospitals, Military"[Mesh]) OR (military hospitals[Title/Abstract])) AND ((breast neoplasms[MeSH Terms])) AND (("2014/02/01"[Date—Publication]: "2024/02/01"[Date—Publication])) AND ((breast cancer[Title/Abstract])) AND ((survival[Title/Abstract]) OR (mortality[Title/Abstract])))	(MM "Military Personnel + ") OR (MH "Hospitals, Military") OR (MH "Military Health") OR (MH "Military Medicine") AND (breast cancer or breast neoplasm or breast carcinoma or breast tumor or breast malignancy [abstract]) AND (survival or mortality or death or outcome [abstract)* *Peer reviewed, English, Feb 2014-Feb 2024, USA geographic subset	(((AB = (Department of Defense or Military Health System)) AND AB = (breast cancer or breast neoplasm or breast carcinoma or breast tumor or breast malignancy)) AND AB = ((survival or mortality or death or outcome)))* *Peer reviewed, English, 1 Feb 2014–1 Feb 2024, USA
Exclusion Criteria	• Published outside of 1 Feb 2014–1 Feb 2024 • Does not include specific quantitative mortality outcomes • Does not compare mortality outcomes by race or does not provide totals to enable comparison by race • Written in a language other than English • Does not include female patients with breast cancer treated in the United States MHS • Non-peer reviewed • Published outside the United States		

*MHS* Military Health System, *Mesh* Medical Subject Headings, *AB* Abstract

### Inclusion and exclusion criteria

Since the goal of this review was ultimately to arrive at a summary effect for odds of mortality comparing Black and White women with BC treated in the MHS, a quantitative outcome associated with mortality and the ability to compare outcomes by race category was necessary for each study included (Table 1). In order to ensure synthesis of the most current research, studies were excluded if they were published outside of 1 February 2014 to1 February 2024. Studies written in a language other than English, published outside the U.S., or published in non-peer reviewed journals or collections were also excluded.

### Study selection, synthesis, and quality assessment

**Fig. 1:**
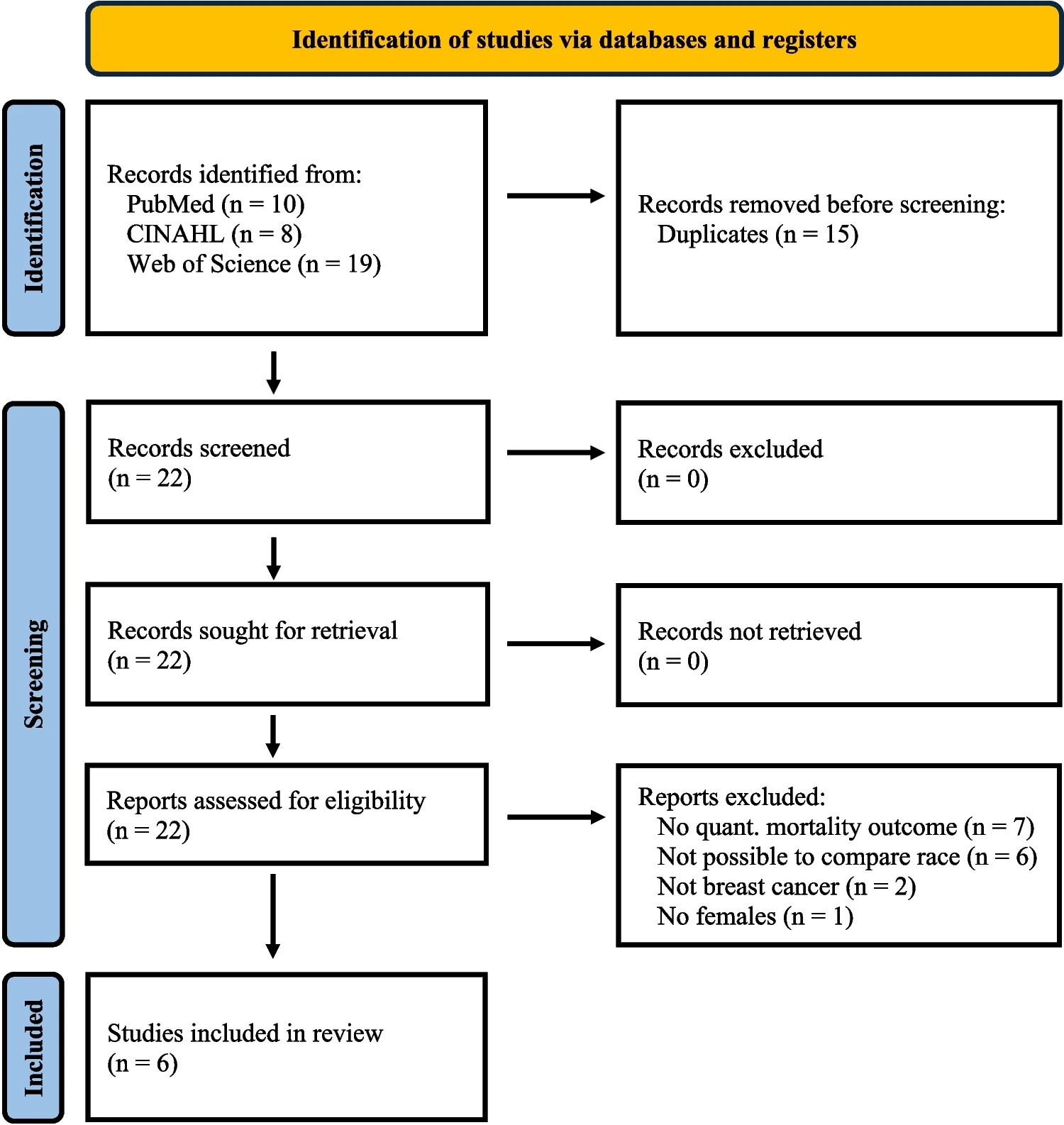
PRISMA flow diagram

Figure 1 includes a detailed flow of the selection process. AJH and ND independently completed the search, study selection, and study quality appraisals. Any disagreements between AJH and ND were adjudicated by YAH. Duplicates were removed using Zotero 6.0 [22]. Articles identified from the databases were initially assessed using title and abstract. Full text reviews were conducted when the title and abstract were insufficient to determine inclusion/exclusion. Reference lists of studies selected for final analysis were also reviewed for additional articles. Data extraction was completed using a summary table which included: source, study title, abstract key points, study design, data source, diagnosis dates included, tumor stages included, population by Black/White, mortality results, 2 × 2 table, and computed mortality odds. Study quality was assessed using the National Institutes of Health, National Heart, Lung, and Blood Institute Study Quality Assessment Tool for Observational Cohort and Cross-Sectional Studies [23].

### Meta-analysis

Odds ratios of mortality comparing Black and White women with BC were calculated by extracting the total number of all-cause deaths to construct 2 × 2 tables [24, 25]. Odds ratios were then converted to log odds for each study, from which standard errors were calculated using Wilson’s effect size calculator [26]. Primary random effects analysis used study specific log odds and restricted maximum likelihood estimator [27] to produce a pooled effect. Additional fixed, or common, effects analysis used inverse variance to generate a pooled effect [28]. Hartung-Knapp-Sidik-Jonkman adjustments were used to account for the small number of studies with differing sample sizes [29]. Sequential sensitivity analyses were performed by first removing the two studies with the largest confidence intervals, and then by removing the most heavily weighted study. Heterogeneity was assessed using tau^2^, I^2^, and Q score [30]. Publication bias was determined visually utilizing a funnel plot, and quantitatively using both Egger’s Test [31] and trim-and-fill [32] methods. Data analyses were conducted using R version 4.3.3 [33], with statistical significance determined using an alpha of 0.05.

## Results

### Overview and data source

Thirty-seven articles were identified across three databases (PubMed: 10, CINAHL: 8, Web of Science: 19). After removing 15 duplicates, 22 studies were assessed for inclusion and 16 were excluded. Six studies were analyzed, which used data from 1987–2013 with samples ranging from 93 to 4,383 for Black and 302 to 24,435 for White women (Table 2). The six retrospective studies used data from the Walter Reed Clinical Breast Cancer Project, DoD cancer registries, the MHS medical data repository, or a combination thereof. No additional articles were identified from reference lists of the six studies included in the analysis. Three articles directly compared mortality risk between races, one study compared mortality for women with Type 2 Diabetes Mellitus (T2DM), and two studies compared DoD patients with matched patient samples from the SEER national database. Two studies reported BC-specific mortality [20, 34], the remaining four studies used all-cause mortality [19, 35–37].

**Table 2:** Summary of 2014 to 2024 studies of mortality comparing Black and White Women with breast cancer treated in the MHS

Source	Data Source	Years of Diagnoses Included	Sample Population (Total Deaths) Black White		Odds Ratio (95% CI) W as ref	Tumor Stages Incl	Key Mortality Findings
Eaglehouse et al. 2019 [19]	DoD Cancer Registry and MDR	1998–2007	988 (115)	3,899 (334)	1.41(1.12–1.76)*	I-III	Black women had higher mortality risk (HR: 1.53; 95% CI, 1.24–1.90*) than White women. Adjusting for demographic, pathologic, and treatment variables attenuated the mortality risk (AHR: 1.21; 95% CI: 0.97–1.52)
Lin et al. 2022 [35]	DoD ACTUR	1987–2013	4,383 (758)	24,435 (3,585)	1.22(1.12–1.33)*	I-IV	Compared to matched SEER patients, both Black (AHR: 0.63, 95% CI: 0.55–0.73*) and White (AHR: 0.78, 95% CI: 0.72–0.83*) women had reduced mortality risk
Lovejoy et al. 2019 [20]	Clinical BC Project (WRNMMC)	2001–2018	271 (12)	628 (24)	1.17(0.57–2.37)	I-III	BC-specific mortality risk was equivalent for Black and White women whether unadjusted (HR: 1.31, 95% CI: 0.65–2.61), or adjusted for demographic and cancer characteristics (AHR: 0.82, 95% CI: 0.35–1.93)
Rizzo et al. 2015 [36]	DoD ACTUR	1996–2008	1,335 (259)	6,548 (1,205)	1.07(0.92–1.24)	I-II	Black and White women had similar odds of mortality (AOR: 0.94, 95% CI: 0.72–1.21) when adjusted for age, tumor stage, recurrence, and surgical procedures
Ru et al. 2017 [34]	Clinical BC Project (WRNMMC)	2001–2010	93 (7)	302 (10)	2.38(0.88–6.43)	I-IV	Compared to SEER matched patients, both Black women (AHR: 0.52, 95% CI: 0.28–0.99*) when adjusted for demographic and tumor characteristics and White women (AHR: 0.59, 95% CI: 0.38–0.92*) when adjusted for demographic characteristics had reduced BC-specific mortality risk
Shao et al. 2018 [37]	DoD Cancer Registry and MDR	1998–2007	1,380 (240)	6,846 (1,011)	1.22(1.04–1.42)*	I-IV	Black and White women with T2DM had equivalent odds of mortality (OR: 1.19, 95% CI: 0.85–1.66), however Black women without T2DM had higher unadjusted odds of mortality (OR: 1.21, 95% CI: 1.01–1.44*) than White women without T2DM

*BC* Breast Cancer, *MHS* Military Health System, *DoD* Department of Defense, *MDR* Military Health System Data Repository, *HR* Hazard Ratio, *AHR* Adjusted Hazard Ratio, *CI* Confidence Interval, *SEER* National Cancer Institute Surveillance, Epidemiology, and End Results Program, *OR* Odds Ratio, *AOR* Adjusted Odds Ratio, *ACTUR* DoD Automated Central Tumor Registry, *WRNMMC* Walter Reed National Military Medical Center, *T2DM* Type II Diabetes. *p < 0.05

### Direct comparison between black and white patients

Three of the six studies directly compared risk of mortality between Black and White patients with BC treated in the MHS [19, 20, 36]. While unadjusted mortality risk varied across studies with direct comparisons, adjusting for treatment, demographic information, and tumor characteristics eliminated significant differences. For example, Eaglehouse et al. found an increased risk of death for Black women compared to White women in unadjusted models (HR: 1.53, 95% CI: 1.24–1.90) that was attenuated and no longer significant once adjusting for demographic, pathologic, and treatment variables (AHR: 1.21, 95% CI: 0.97–1.52) [19].

### Comparisons among patients with T2DM

One study focused on whether a T2DM diagnosis, before or after a BC diagnosis, was associated with higher risk of mortality when stratified by race [37]. Shao et al. noted that Black patients with T2DM diagnosed at or after a BC diagnosis had significantly greater mortality risk (AHR: 2.05, 95% CI: 1.36–3.07) compared to their counterparts without T2DM when adjusting for potential confounders [37]. No significant differences in mortality were found in similar comparisons among White patients with and without a T2DM diagnosis [37]. Using 2 × 2 tables from the study population to determine unadjusted odds of mortality, Black and White women with a T2DM diagnosis-whether before or after a BC diagnosis-had equivalent unadjusted odds of mortality (OR: 1.19, 95% CI: 0.85–1.66), however Black women without a T2DM diagnosis had higher unadjusted odds of mortality (OR: 1.21, 95% CI: 1.01–1.44) than White women without T2DM.

### Comparison with U.S. general population

In two studies that compared both Black and White women with BC treated in the MHS to matched SEER samples, both Black and White women treated in the MHS had significantly lower risk of mortality when adjusted for demographic, tumor characteristics, and treatment type [34, 35]. Ru et al. noted that the significant reduction in risk of mortality between White DoD patients compared to White SEER matched patients became insignificant when tumor characteristics were added to the adjustments for demographic variables [34]. Lin et al. found that Black patients had substantially lower risk of mortality than their matched SEER counterparts (AHR: 0.63, 95% CI: 0.55–0.73), when adjusted for matching characteristics, tumor characteristics, and treatment type [35].

### Meta-analysis

**Fig. 2:**
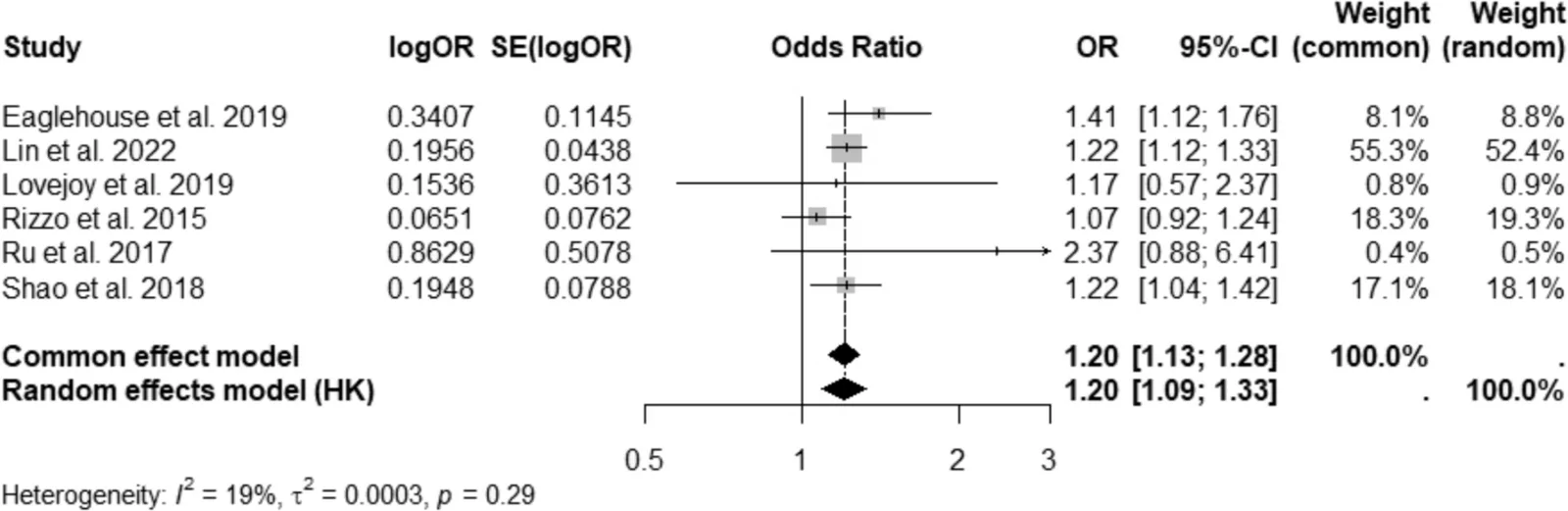
Odds of mortality for Black, compared to White, women with breast cancer treated in the MHS from studies published 2014 to 2024

Figure 2 includes the unadjusted odds of mortality for Black women compared to White women treated for BC within the MHS from studies published between 2014 and 2024. After calculating individual odds for each study, three had significantly higher odds of mortality for Black women, while the effect did not reach statistical significance in the remaining three studies. In random effects models, Black women had significantly higher unadjusted summary odds of mortality (OR: 1.20, 95% CI: 1.09–1.33) compared to white women with BC treated in the MHS. Fixed effects (OR: 1.20, 95% CI: 1.13–1.28) analyses produced nearly identical pooled odds indicating minimal between-study heterogeneity. The random effects model odds remained similar (OR: 1.20, 95% CI: 1.05–1.37) after removing the two studies with the largest confidence intervals. Since weights used to calculate the summary effect may have minimized the impact of removing the two studies with the largest confidence intervals, we also tested the summary effect after removing only the most heavily weighted study. After removing the most heavily weighted study, the pooled effect of the random effects model failed to reach statistical significance as the confidence interval expanded (OR: 1.21, 95% CI: 1.00–1.47).

### Heterogeneity and publication bias

**Fig. 3:**
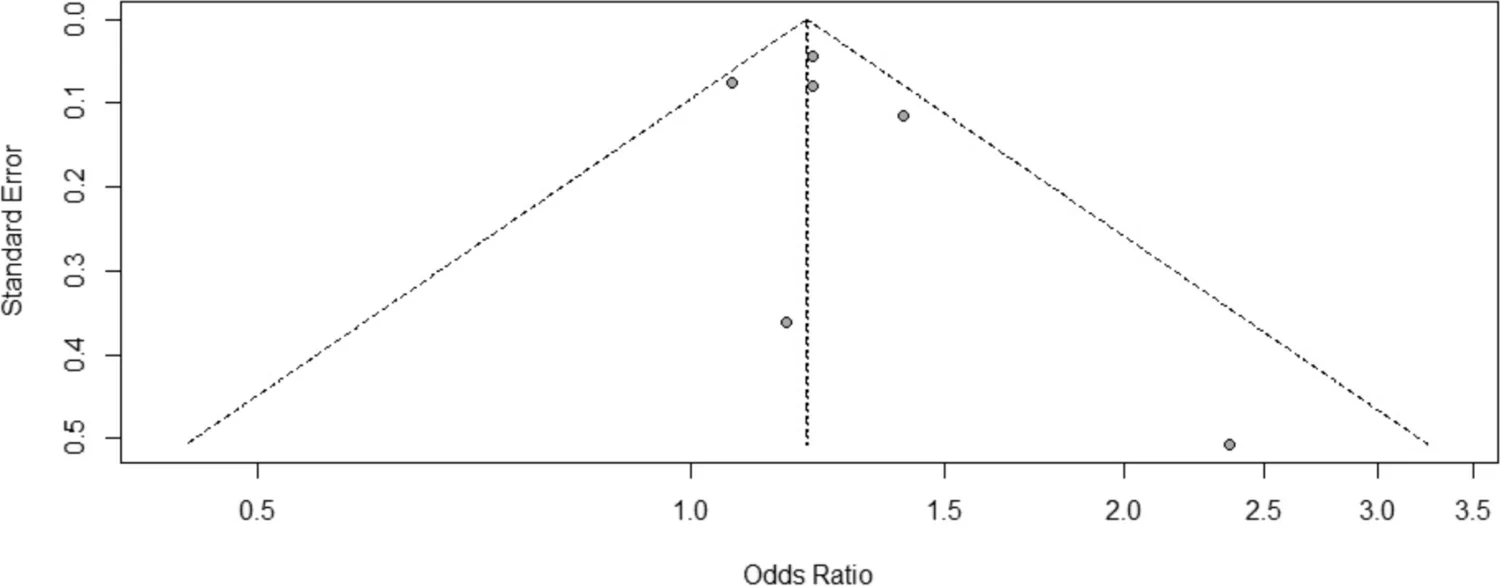
Publication bias of 2014 to 2024 studies of mortality comparing Black and White Women with breast cancer treated in the MHS

The six studies are fairly homogeneous, as measured by Tau^2^ (0.0003), I^2^ (19%), and Q (*p* = 0.289) [30]. Specifically, the relatively small between-study heterogeneity (I^2^ = 19%) resulted in almost identical pooled effects, whether computed using random or fixed effects. In both sub analyses removing the two studies with the largest confidence intervals, and removing the most heavily weighted study, low heterogeneity persisted across all measures. Figure 3 shows the publication bias of the studies using a funnel plot. The plot and additional analyses including the Egger’s Test (*p* = 0.439) and trim-and-fill (OR: 1.20, 95% CI: 1.09–1.32) suggest a very low likelihood of publication bias in the analysis [31, 32]. Using the same measures of publication bias for sub analyses produced comparably low likelihood of publication bias, although when the most heavily weighted study was removed, the summary odds of the trim-and-fill method became insignificant (OR: 1.14, 95% CI: 0.93–1.40) as in the main model.

### Study quality assessment

Each study was rated “good” on a scale of “good”, “fair”, or “poor” using the National Institutes of Health, National Heart, Lung, and Blood Institute Study Quality Assessment Tool for Observational Cohort and Cross-Sectional Studies [23]. A minimum of ten years of data was used for each study population, and exclusion/inclusion criteria were consistently applied. Outcomes and exposures were clearly defined in each paper. For two studies which directly compared DoD populations to SEER matched samples, no more than a 1:5 ratio was used for comparison groups. Confounders were also clearly defined in each study.

## Discussion

A review of the last ten years of empirical research comparing mortality risk between Black and White women treated in the MHS for BC showed varied results. Adjusting for demographic and cancer characteristics in three studies which directly compared Black and White women eliminated significant differences in mortality risk [19, 20, 36]. Black and White women with BC and T2DM had equivalent odds of mortality, while a comparison of women without T2DM in the same study found Black women had higher unadjusted odds of mortality than White women [37]. Compared to matched SEER patients, both Black and White women treated in the MHS had reduced mortality risk [34, 35], although the reduced risk became statistically insignificant for White patients when tumor characteristics were added to previous adjustments for demographic variables in one study [34]. In the current meta-analysis across all six papers, using 2 × 2 tables resulted in a significantly higher summary odds of mortality for Black patients compared to White patients.

The results of the meta-analysis suggest that while access to health care may play a part, equal access may not completely eliminate racial disparities in mortality risk among BC patients [4, 5]. More specifically, the lack of disparities in screening [17] and increased survival in patients with BC treated in the MHS compared to the U.S. general population [34, 35] point to the value of future research into causes for such persistent racial disparities in all-cause mortality for patients with BC. The significant differences in mortality between Black and White women with BC in the meta-analysis indicate more work is needed to reduce disparities. With the most recent research on direct comparison of racial disparities in mortality being from 2019, the DoD and its beneficiaries would benefit from updated analysis using multiple models with varying confounders and BC-specific mortality. In addition to the noted benefits of early screening and access to a wide spectrum of treatment modalities at little or no cost, future research parsing out the specific aspects of access that reduce disparities in mortality would improve understanding on what remains to be done to achieve health equity.

Recent research into equity within healthcare highlights the need for additional investigation into social stress, environmental exposures, and genetic factors influencing cancer outcomes [2, 3]. While access is clearly a key factor impacting racial disparities in outcomes for patients with BC, research into these additional factors may help close the gap in mortality risk between Black and White women treated in the U.S.. Krieger et al. noted a significant difference in odds of certain types of BC based on place of birth for Black and White women [38]. Lichtenstein et al. showed in a multinational twin study that hereditary components’ contribution to breast cancer was only 27%, resulting in the authors attributing breast cancer primarily to environmental factors [39]. Bigatti et al. found that, in a small cohort, treatment adherence may be impacted by familial, social, work-related, or other factors [40]. Yee et al. showed how distress levels among Black women receiving BC treatment was associated with lower adherence to chemotherapy [41]. Jackson et al. found that stressors experienced by Black women may result in increased morbidity or mortality compared to White women [42]. Building on this idea of stressors and the connection with health, specifically BC, Mohan et al.’s narrative review found correlation between stress and BC through endocrine pathways [43]. Barber et al. found that poor sleep health was associated with increased odds of BC in Black, but not White women [44]. These examples are just a few of the recent studies on social determinants of health and risk factors other than access that may play critical roles in racial disparities in mortality among patients with BC. Future work to identify contributing factors to disparities and develop specific, targeted interventions for other areas of health will help ensure equal access equates to health equity.

To the best of our knowledge, this is the first review of its kind comparing mortality between Black and White women treated for BC in the MHS. Another strength of this review is the quantitative assessment of pooled mortality risk, using six independent studies published over the last ten years. This review also has several limitations. Only three of the six studies contained direct comparison of mortality between Black and White patients. Due to the inconsistency of confounders and timeframes with the odds and hazard ratio calculations, we were unable to quantitatively compare adjusted mortality risk, which may have modified our pooled estimates. Lastly, the differences inherent to mortality in terms of observation timeline were not accounted for in this research as all-cause death was used across studies with differing timeframes. As the focus of this paper was disparities in mortality, these time specific differences were assumed to apply equally to both Black and White women in each study.

## Conclusions

While equal access may limit the effect of racial disparities in mortality between Black and White women with BC, equal access alone may not eliminate these inequities. Additional research is needed to understand the racial disparities in mortality among patients with BC and targeted interventions are needed to reduce such disparities.

## Data Availability

Table 1 includes all search terms used in this systematic review and meta-analysis.

## Declarations

### Disclaimer

The contents of this publication are the sole responsibility of the authors and do not reflect the views, assertions, opinions or policies of the Uniformed Services University of the Health Sciences, the Henry M. Jackson Foundation for the Advancement of Military Medicine, Inc., the Department of Defense, or the Departments of the Army, Navy, or Air Force., or any other agency of the U.S. Government. Mention of trade names, commercial products, or organizations does not imply endorsement by the U.S. Government.

### Institutional clearance

The Uniformed Services University of the Health Sciences Office of External Affairs granted clearance to submit the manuscript for publication.

## References

[1] Siegel RL, Giaquinto AN, Jemal A. “Cancer statistics, 2024. CA: A Cancer J Clin. 2024;74(1):12–49. 10.3322/caac.21820. 38230766

[2] Minas TZ, Kiely M, Ajao A, Ambs S. An overview of cancer health disparities: new approaches and insights and why they matter. Carcinogenesis. 2020;42(1):2–13. 10.1093/carcin/bgaa121. PMC7717137 33185680

[3] Williams DR, Mohammed SA, Leavell J, Collins C. Race, socioeconomic status and health: complexities, ongoing challenges and research opportunities. Ann N Y Acad Sci. 2010;1186:69–101. 10.1111/j.1749-6632.2009.05339.x. PMC3442603 20201869

[4] Ismail J, Hyuna S, Ahmedin J. The emergence of the racial disparity in U.S. breast-cancer mortality. N Engl J Med. 2022;386(25):2349–52. 10.1056/NEJMp2200244. 35713541

[5] Jatoi I, Anderson WF, Rao SR, Devesa SS. Breast cancer trends among black and white women in the United States. J Clin Oncol. 2005;23(31):7836–41. 10.1200/JCO.2004.01.0421. 16258086

[6] J. Hale, N. Hong, B. Hopkins, S. Lyons, E. Molloy, and null The Congressional Budget Office Coverage Team. “Health insurance coverage projections for the US population and sources of coverage, by age, 2024–34. Health Aff (Millwood). 2024;43(7):922–932. 10.1377/hlthaff.2024.00460. 38889370

[7] Castaldi M, Smiley A, Kechejian K, Butler J, Latifi R. Disparate access to breast cancer screening and treatment. BMC Womens Health. 2022;22:249. 10.1186/s12905-022-01793-z. PMC9219222 35733197

[8] Jemal A, et al. Factors that contributed to black-white disparities in survival among nonelderly women with breast cancer between 2004 and 2013. J Clin Oncol. 2018;36(1):14–24. 10.1200/JCO.2017.73.7932. 29035645

[9] Ganguly AP, Baker KK, Redman MW, McClintock AH, Yung RL. Racial disparities in the screening mammography continuum within a heterogeneous health care system. Cancer. 2023;129(S19):3171–81. 10.1002/cncr.34632. 37691529

[10] Elmore JG, Nakano CY, Linden HM, Reisch LM, Ayanian JZ, Larson EB. Racial inequities in the timing of breast cancer detection, diagnosis, and initiation of treatment. Med Care. 2005;43(2):141–8. 10.1097/00005650-200502000-00007. 15655427

[11] Bach PB, Schrag D, Brawley OW, Galaznik A, Yakren S, Begg CB. Survival of blacks and whites after a cancer diagnosis. JAMA. 2002;287(16):2106–13. 10.1001/jama.287.16.2106. 11966385

[12] “Evaluation of the TRICARE program: FY 2022 report to congress,” Feb. 2022. [Online]. Available: https://www.health.mil/Reference-Center/Reports/2022/10/07/Evaluation-of-the-TRICARE-Program-FY-2022-Report-to-Congress. Accessed 15 Feb 2024.

[13] “TRICARE Health Plan Costs,” Department of Defense, Mar. 2024. [Online]. Available: https://tricare.mil/Publications/Costs/costs_fees. Accessed 24 Apr 2024

[14] Department of Defense, “Incidence of breast cancer among members of the armed forces services on active duty,” report to congress, Mar. 2024. [Online]. Available: https://www.health.mil/Reference-Center/Reports/2024/03/05/Study-on-Incidence-of-Breast-Cancer-Among-ADSMs. Accessed 23 Apr 2024.

[15] Lovejoy LA, Shriver CD, Ellsworth RE. “Cancer Incidence and Etiology in the Active Duty Population of U.S. Military. Mil Med. Oct. 2022;usac297. 10.1093/milmed/usac297. 36239575

[16] Zhu K, et al. Cancer incidence in the U.S. military population: comparison with rates from the SEER program. Cancer Epidemiol Biomarkers Prev. 2009;18(6):1740–5. 10.1158/1055-9965.EPI-09-0041. PMC2780333 19505907

[17] Manjelievskaia J, Brown D, Shao S, Hofmann K, Shriver CD, Zhu K. Breast cancer treatment and survival among department of defense beneficiaries: an analysis by benefit type and care source. Mil Med. 2018;183(3–4):e186–95. 10.1093/milmed/usx031. PMC6927891 29365174

[18] Bytnar JA, et al. The impact of mammography screening guideline changes among women serving in the U.S. military. Mil Med. 2020;185(11–12):e2088–96. 10.1093/milmed/usaa176. PMC9891101 32803239

[19] Eaglehouse YL, Georg MW, Shriver CD, Zhu K. Racial differences in time to breast cancer surgery and overall survival in the US military health system. JAMA Surg. 2019;154(3):e185113. 10.1001/jamasurg.2018.5113. PMC6439631 30673075

[20] Lovejoy LA, Eaglehouse YL, Hueman MT, Mostoller BJ, Shriver CD, Ellsworth RE. Evaluation of surgical disparities between african american and european american women treated for breast cancer within an equal-access military hospital. Ann Surg Oncol. 2019;26(12):3838–45. 10.1245/s10434-019-07706-z. 31410609

[21] Page MJ, et al. The PRISMA 2020 statement: an updated guideline for reporting systematic reviews. BMJ. 2021;372:n71. 10.1136/bmj.n71. PMC8005924 33782057

[22] Center for History and New Media, “Zotero 6.0.” George Mason University. [Online]. Available: https://www.zotero.org/. Accessed 28 Mar 2024.

[23] “Study Quality Assessment Tools | NHLBI, NIH.” [Online]. Available: https://www.nhlbi.nih.gov/health-topics/study-quality-assessment-tools. Accessed 23 Apr 2024.

[24] Sauerbrei W, Blettner M. Interpreting Results in 2 × 2 Tables. Dtsch Arztebl Int. 2009;106(48):795–800. 10.3238/arztebl.2009.0795. PMC2797398 20038982

[25] Hirji KF, Fagerland MW. Calculating unreported confidence intervals for paired data. BMC Med Res Methodol. 2011;11:66. 10.1186/1471-2288-11-66. PMC3115921 21569392

[26] Wilson DB. “Practical meta-analysis effect size calculator (Version 2023.11.27).” [Online]. Available: https://www.campbellcollaboration.org/escalc/index.php. Accessed 28 Mar 2024.

[27] Patterson HD, Thompson R. Recovery of inter-block information when block sizes are unequal. Biometrika. 1971;58(3):545–54. 10.1093/biomet/58.3.545.

[28] Deeks J, Higgins J, Altman D. Analyzing data and undertaking meta-analyses. In: Higgins J, Green S, editors. Cochrane Handbook for Systematic Reviews of Interventions. Oxford, England: Wiley-Blackwell; 2008.

[29] IntHout J, Ioannidis JPA, Borm GF. The Hartung-Knapp-Sidik-Jonkman method for random effects meta-analysis is straightforward and considerably outperforms the standard DerSimonian-Laird method. BMC Med Res Methodol. 2014;14:25. 10.1186/1471-2288-14-25. PMC4015721 24548571

[30] Higgins JPT, Thompson SG. Quantifying heterogeneity in a meta-analysis. Stat Med. 2002;21(11):1539–58. 10.1002/sim.1186. 12111919

[31] Egger M, Smith GD, Schneider M, Minder C. “Bias in meta-analysis detected by a simple, graphical test. BMJ. 1997;315(7109):629–34. 10.1136/bmj.315.7109.629. PMC2127453 9310563

[32] Duval S, Tweedie R. Trim and fill: A simple funnel-plot-based method of testing and adjusting for publication bias in meta-analysis. Biometrics. 2000;56(2):455–63. 10.1111/j.0006-341x.2000.00455.x. 10877304

[33] “R Core Team (2024). _R: A Language and Environment for Statistical Computing_.,” R Foundation for Statistical Computing, Vienna, Austria. [Online]. Available: https://www.R-project.org. Accessed 17 Jan 2024

[34] Ru Y, et al. Comparative survival analysis of invasive breast cancer patients treated by a U.S. military medical center and matched patients from the U.S. general population. Mil Med. 2017;182(11):e1851–8. 10.7205/MILMED-D-17-00097. 29087852

[35] Lin J, Hu H, Shriver CD, Zhu K. Survival among breast cancer patients: comparison of the U.S. military health system with the surveillance, epidemiology and end results program. Clin Breast Cancer. 2022;22(4):e506–16. 10.1016/j.clbc.2021.11.010. 34961733

[36] Rizzo JA, Sherman WE, Arciero CA. Racial disparity in survival from early breast cancer in the department of defense healthcare system. J Surg Oncol. 2015;111(7):819–23. 10.1002/jso.23884. 25711959

[37] Shao S, et al. Diabetes and overall survival among breast cancer patients in the u.s. military health system. Cancer Epidemiol Biomarkers Prev. 2018;27(1):50–7. 10.1158/1055-9965.EPI-17-0439. PMC5808886 29097445

[38] Krieger N, Jahn JL, Waterman PD. Jim Crow and estrogen-receptor-negative breast cancer: US-born black and white non-Hispanic women, 1992–2012. Cancer Causes Control. 2017;28(1):49–59. 10.1007/s10552-016-0834-2. 27988896

[39] Paul L, et al. Environmental and heritable factors in the causation of cancer — analyses of cohorts of twins from Sweden, Denmark, and Finland. N Engl J Med. 2000;343(2):78–85. 10.1056/NEJM200007133430201. 10891514

[40] Bigatti SM, Weathers T, Hayes L, Daggy J. “Challenges experienced by black women with breast cancer during active treatment: relationship to treatment adherence. J Racial Ethn Health Disparities. Feb. 2023;1–12. 10.1007/s40615-023-01537-1 PMC9945826 36811760

[41] Yee MK, Sereika SM, Bender CM, Brufsky AM, Connolly MC, Rosenzweig MQ. Symptom incidence, distress, cancer-related distress, and adherence to chemotherapy among African American women with breast cancer. Cancer. 2017;123(11):2061–9. 10.1002/cncr.30575. 28199006

[42] Jackson JS, Knight KM, Rafferty JA. Race and unhealthy behaviors: chronic stress, the HPA Axis, and physical and mental health disparities over the life course. Am J Public Health. 2010;100(5):933–9. 10.2105/AJPH.2008.143446. PMC2853611 19846689

[43] Mohan A, Huybrechts I, Michels N. Psychosocial stress and cancer risk: a narrative review. Eur J Cancer Prev. 2022;31(6):585–99. 10.1097/CEJ.0000000000000752. 35352705

[44] Barber LE, McCullough LE, Johnson DA. Eyes wide open: sleep as a potential contributor to racial and ethnic disparities in cancer. Cancer Epidemiol Biomark Prev. 2024;33(4):471–9. 10.1158/1055-9965.EPI-23-1117. PMC10990828 38270540

